# Polyaniline and Polyaniline-Based Materials as Sorbents in Solid-Phase Extraction Techniques

**DOI:** 10.3390/ma15248881

**Published:** 2022-12-12

**Authors:** Ireneusz Sowa, Magdalena Wójciak, Katarzyna Tyszczuk-Rotko, Tomasz Klepka, Sławomir Dresler

**Affiliations:** 1 Department of Analytical Chemistry, Medical University of Lublin, Chodźki 4a, 20-093 Lublin, Poland; 2 Institute of Chemical Sciences, Faculty of Chemistry, Maria Curie-Skłodowska University in Lublin, 20-031 Lublin, Poland; 3 Department of Technology and Polymer Processing, Faculty of Mechanical Engineering, Lublin University of Technology, Nadbystrzycka 36, 20-618 Lublin, Poland; 4 Department of Plant Physiology and Biophysics, Institute of Biological Science, Maria Curie-Skłodowska University, Akademicka 19, 20-033 Lublin, Poland

**Keywords:** PANI, solid-phase extraction, polyaniline based composites, sample pretreatment

## Abstract

Polyaniline (PANI) is one of the best known and widely studied conducting polymers with multiple applications and unique physicochemical properties. Due to its porous structure and relatively high surface area as well as the affinity toward many analytes related to the ability to establish different types of interactions, PANI has a great potential as a sorbent in sample pretreatment before instrumental analyses. This study provides an overview of the applications of polyaniline and polyaniline composites as sorbents in sample preparation techniques based on solid-phase extraction, including conventional solid-phase extraction (SPE) and its modifications, solid-phase microextraction (SPME), dispersive solid-phase extraction (dSPE), magnetic solid-phase extraction (MSPE) and stir-bar sorptive extraction (SBSE). The utility of PANI-based sorbents in chromatography was also summarized. It has been shown that polyaniline is willingly combined with other components and PANI-based materials may be formed in a variety of shapes. Polyaniline alone and PANI-based composites were successfully applied for sample preparation before determination of various analytes, both metal ions and organic compounds, in different matrices such as environmental samples, food, human plasma, urine, and blood.

## 1. Introduction

Polyaniline (PANI) is one of the best known and widely studied conducting polymers with multiple applications in many fields, including physics, electronics, energy storage, optics, materials, biomedical science, and many others. This interesting material with unique physicochemical properties is characterized by good conductivity, stability, easy synthesis, redox properties, porous structure (and hence a relatively large surface area), and the affinity toward many analytes related to its ability to establish different types of interactions [1,2,3]. 

Some of the aforementioned features make PANI a promising material for sample pretreatment aiming at recovery of analytes from samples, removal of interferents that may disturb the analysis, and/or concentration of the analyte before instrumental determination. It is an essential step of the analytical procedure as it may be a source of about 30% of experimental errors. Various approaches for sample pretreatment have been developed, including simple filtration, evaporation, and resolubilization. However, techniques based on solid-phase extraction have the greatest importance in laboratory practice. The procedure can be performed in different modes such as solid-phase microextraction (SPME), dispersive solid-phase extraction (dSPE), magnetic solid-phase extraction (MSPE), stir-bar sorptive extraction (SBSE), etc. [4,5]. The choice of the variant depends on the physicochemical characteristics and concentration of the compound of interest, the matrix composition, and the further analytical procedures [6,7,8].

**Figure 1:**
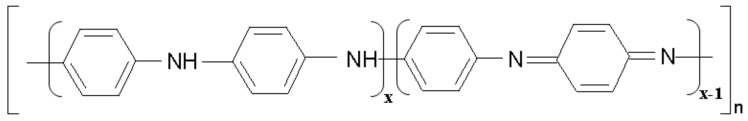
General structure of polyaniline.

Polyaniline (PANI) alone (Figure 1), its substituted derivatives, and more frequently, complex composites comprising inorganic or organic components, have become attractive materials for sample preparation before the instrumental analysis of various analytes in different matrices.

Our work summarizes the applications of PANI and PANI composites in sample pretreatment techniques based on solid-phase extraction.

It should also be noted that the sorption capacity of polyaniline and polyaniline composites is also utilized in the processes of purification/removal of various chemicals, including metal ions or dyes. However, the detailed application of PANI composites in these fields was described previously [9,10,11,12,13]; hence, this topic was excluded from our study. Moreover, it does not cover a typical sample pretreatment procedure for analytical purposes. 

## 2. General Information

There are many review papers describing different methods of synthesis, detailed structure, and physicochemical properties of polyaniline. Therefore, this section is focused only on the basic data that may be important from the point of view of the application of PANI as a sorbent in separation techniques.

### 2.1. Synthesis

Polyaniline can be formed in both aqueous and non-aqueous media, and the synthesis process is relatively easy. In the chemical synthesis of PANI, three types of reactants are required, including aniline as the main substrate, an oxidant, and acid ensuring the formation of water-soluble aniline salt. The polymerization process is initialized by addition of an oxidizing agent to the solution of aniline. Ammonium persulfate ((NH_4_)_2_S_2_O_8_), sodium vanadate (NaVO_3_), cerium sulfate (Ce(SO_4_)_2_), hydrogen peroxide (H_2_O_2_), potassium iodate (KIO_3_), or potassium dichromate (K_2_Cr_2_O_7_) may be used as an oxidizer; however, ammonium persulfate ((NH_4_)_2_S_2_O_8_) is the most common agent. The reaction takes places in an acidic medium (pH ≤ 3) with the presence of hydrochloric acid or sulfuric acid at a low temperature in an ice bath. Polymerization takes from a few to several hours, and after that, PANI is separated by filtration and is rinsed with deionized water followed by alcohol and acetone or other organic solvents to ensure that non-reactive materials have been completely removed. In situ polymerization of aniline directly on the carrier is the most convenient way for preparation of PANI-based sorbents [1,2,3]. A large variety of dopants and additives can be added during the synthesis, which provides materials with new synergistic or complementary properties. 

### 2.2. Structure and Physicochemical Properties of PANI

**Figure 2:**
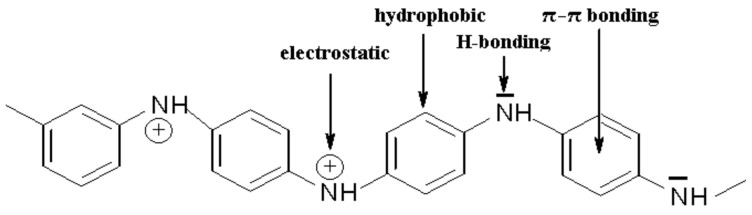
Types of interactions on polyaniline.

Polyaniline has aromatic rings combined with nitrogen atoms and a system of conjugated single and double bonds. Such a chemical structure allows diverse interactions between the PANI layer and different analytes. Nitrogen atoms are able to form hydrogen bonds and, due to the presence of aromatic rings, hydrophobic π–π interactions may occur. In addition, PANI has charged ionic groups capable of electrostatic binding with the anionic form (Figure 2). 

**Figure 3:**
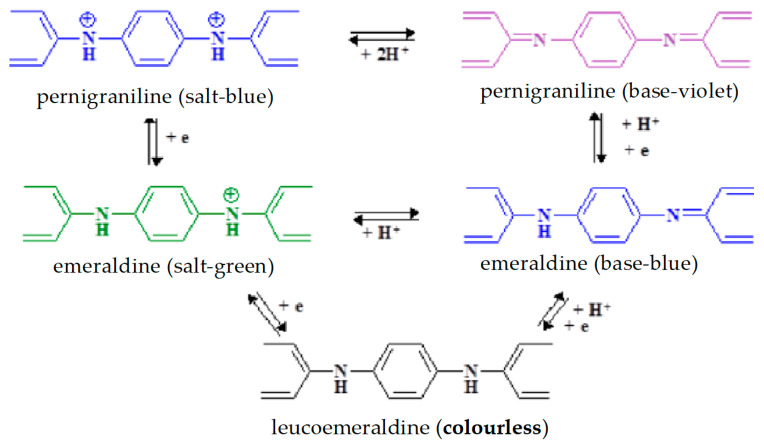
Different forms of polyaniline depending on acid–base conditions and oxidation states.

PANI can be found in various forms with different colors depending on acid–base conditions and oxidation states: leucoemeraldine (colorless), emeraldine (salt-green/base-blue), and pernigraniline (salt-blue/base-violet). Pernigraniline is the fully oxidized form, emeraldine is the half-oxidized form (the most stable), and lecomeraldine is the reduced form of PANI. In acidic conditions, the PANI hydrophobic non-conducting base is converted to hydrophobic conducting salt (Figure 3) [1,2,3].

It should be also noted that polyaniline is stabile both in strongly alkaline and strongly acidic conditions and has high-long term thermal stability enabling processing up to 250–350 °C [14,15].

## 3. Application of Polyaniline and Polyaniline Composites in Separation Techniques Based on Solid-Phase Extraction

Solid-phase extraction (SPE) is the most commonly used technique to isolate compounds from liquid matrices because it is rapid, simple, and repeatable and has a wide range of applications from environmental to biological samples. Polarity of the sorbent and physico-chemical properties of analytes should be taken into account during optimization of the procedure. SPE can be used to concentrate and isolate the analyte from interferents or to retain an interfering matrix. The process can be carried out using various modes and modifications that differ in the placement of the sorbent and the way of loading the sample.

### 3.1. Conventional Solid-Phase Extraction (SPE) and Its Modifications

In this technique, usually a portion of the sorbent (from 100 mg to even 10 g) is placed in glass or polypropylene (PE) tubes with a volume from 1 to 60 mL with protective PE frits preventing movement of the bed. After activation and loading the sample, the SPE filling is treated with solvents with different elution strengths. The compound of interest may be retained in the sorbent, which is further washed to remove pollutants, and finally the analyte is eluted using a few mL of the solvent. In another approach, the analyte passes through the bed and the matrix remains in the SPE filling [7,8]. 

There are some reports describing the applications of polyaniline or polyaniline-covered materials in the SPE technique. For example, Bagheri et al. used polyaniline nanowires to isolate pesticides and phenol derivatives and, in the form of a sorbent-packed syringe, for extraction of triazine, organochlorine, and organophosphorous pesticides from aqueous samples [16,17,18,19]. Moreover, PANI deposited electrochemically on a stainless steel mesh was exploited to isolate polycyclic aromatic hydrocarbons (PAHs) from real water samples [20]. In turn, Sowa et al. used silica (Si) as a carrier and covered Si particles with a polyaniline film by in situ polymerization of aniline directly on silica. Such a stationary phase was thermally stabile and resistant to pH changes in both acidic and basic conditions [21,22]. Si-PANI was successfully applied for pretreatment of water before determination of inorganic ions [23] and for preparation of plant samples before analysis of acidic [24] and alkaline compounds [25]. Modification of Si-PANi with silver nanoparticles and Si-PANI impregnated with Acid Alizarin Violet N was used for purification of water from some heavy metal ions [26]. Combinations of PANI with different materials, including polyacrylonitrile, poly(styrene-divinyl benzene), multi-walled carbon nanotubes (MWCNT), chitosan, and many others were also exploited in SPE [27,28,29,30,31]. 

A two-component system consisting of PANI with a copolymer of tetrafluoroethylene and vinyliden fluoride (FP-PANI) and a layer of alginate spheres was applied for one-step isolation of DNA from soil extracts. Such filling effectively bonded proteins from the soil lysate and did not retain DNA [32].

A PANI-based sorbent was also used in micro-SPE (μSPE), a miniaturized variant of SPE, in which the sample solution is pumped through a cartridge packed with extractive material [33], and in microextraction in a packed syringe (MEPS) when a few mg of sorbent were inserted inside the syringe between two polyethylene filters [34]. 

Pipette-tip solid-phase extraction (PT-SPE), i.e., another variant of SPE, on sorbent modified with PANI was used to extract some flavonoids from *Epipremnum aureum* [30], sulfonamide from milk and honey samples [35], and fluoxetine/norfluoxetine from plasma [36]. A recent development in SPE are the microfluidic devices which fit the modern trends for searching for new, green, cost-effective, miniaturized, and fast sample preparation techniques. A microfluidic chip based on a polyurethane–PANI composite was fabricated by Farahani et al. [37] and successfully applied to isolate some alkaloids from biological fluids. 

Table 1 summarizes the application of polyaniline and polyaniline-based material as sorbents in the SPE technique.

### 3.2. Solid-Phase Microextraction (SPME)

Solid-phase microextraction (SPME) is a solvent-free sample preparation technique requiring only small amounts of an adsorbent for the extraction of analytes from the sample matrix. SPME was elaborated in 1990 by Pawliszyn and Arthur [43] as an alternative to SPE and is regarded as a green sample preparation technique. Nowadays, it is widely applied in various fields, e.g., in food, environmental, and biological investigations [4,6]. In SPME, the extraction phase is usually applied in the form of a monolithic fiber or a thin layer immobilized on the carrier (wire or fiber) using the sol-gel process or electrodeposition. The separation process in SPME can be carried out using different modes, including direct immersion SPME (DI-SPME), headspace SPME (HS-SPME), and membrane extraction [5,6]. In the case of polyaniline-based sorbents, the DI and HS modes are the most useful. Moreover, it should be noted that PANI alone was rarely used in the SPME technique; more often, it was a component of different nanomaterials covering platinum or stainless steel wire. The deposition of PANI on the carrier can be performed in two ways: by electropolymerization (electrodeposition) or by chemical oxidation; however, the former one is the most common method for coating SPME fibers.

#### 3.2.1. Direct-Immersion Solid-Phase Microextraction (DI-SPME) 

In the direct mode of SPME, the fiber is immersed directly into a small volume of the liquid sample and the extraction process is often accompanied by agitation to support movement of the analyte towards the fiber [4]. Polyaniline deposited on platinum or gold was used as a sorption material to extract benzaldehyde from pharmaceutical formulations as well as phenol, its derivatives, anatoxin-a, and polycyclic aromatic hydrocarbons from water samples [44,45,46,47,48]. In turn, stainless steel wire covered with PANI was used for extraction of phthalates, chloro- and nitrobenzenes, and organochlorine pesticides from water samples [49,50,51]. Other materials, e.g., titania nanotubes, polydimethylsiloxane (PDMS), and basalt fibers were also found to be useful as carriers for PANI in the SPME technique [52,53,54]. 

As mentioned above, pure polyaniline has minor significance in the SPME technique; however, PANI is a readily used and desired component of numerous composites or is doped with various additives, such as fluorinated organic acid. For example, fluorinated PANI was employed to isolate polycyclic aromatic hydrocarbons and polychlorinated biphenyls from water [54,55], and PANI doped with PEG and polydimethylsiloxane effectively isolated phenols from water samples [56]. Composites consisting of PANI with different forms of carbon, including multi-walled carbon nanotubes, graphene, and graphene oxide, are also often fabricated [57,58,59]. 

Table 2 presents the application of polyaniline and polyaniline composites in DI-SPME.

#### 3.2.2. Headspace Solid-Phase Microextraction (HS-SPME)

In HS-SPME, the fiber is placed in the headspace of a sample solution in a vessel and analytes are absorbed/adsorbed onto the sorbent from the gas phase. The technique is mainly used for volatiles that are further analyzed by gas chromatography [4]. There are some reports describing the application of pure PANI in HS-SPME, e.g., polyaniline deposited on a gold wire was successfully applied to extract aliphatic alcohols, phenol, and 4-chlorophenol from gaseous samples [82,83], and PANI on stainless steel was used for isolation of benzene derivatives from water and organoarsenic and organophosphorus compounds in soil samples [55,84]. However, PANI was much more frequently used as a component of composites with various materials, including ionic liquids, carbon nanotubes, montmorillonite, polypyrrole, etc. (Table 3). It is worth mentioning that, in SPME, the sorption material usually covers the fiber although different solutions can be applied as well. Gholivand and Abolghasemi placed highly porous polyaniline combined with hexagonally ordered silica on the interior surface of a hollow stainless steel needle, and sampling was carried out by active drawing a specific volume of the gaseous or aqueous mixture. This facilitated extraction of polycyclic aromatic hydrocarbons from water followed by GC-MS analysis [62]. A similar approach was applied by Ghiasvand et al. to isolate polycyclic aromatic hydrocarbons from polluted soil samples with the use of a polyaniline/multi-wall carbon nanotube composite [85]. Headspace in-needle microextraction using a stainless steel needle coated with a polyaniline layer was also used to remove phthalates from water [86].

In turn, in a study conducted by Bagheri and Aghakhani, a composite of polyaniline with nylon-6 was electrospun to form a fibrous sheet with nano-scale dimensions and was applied for the headspace adsorptive microextraction of selected chlorobenzenes (CBs). The nanofiber sheet was located inside a metallic cylinder and exposed to the gaseous phase while heating the sample solution in a circulating water bath [87].

### 3.3. Dispersive Solid-Phase Extraction (dSPE)

Dispersive solid-phase extraction (dSPE) has gained popularity since 2003 when it was first time described by Anastassiades et al. [102]. In this technique, the sorbent is placed directly into the liquid sample solution, which is followed by vigorous shaking and centrifugation. [103]. There are only some reports describing classic dSPE with the use of polyaniline-based material. Sowa at al. applied dSPE with polyaniline-covered silica to isolate triterpenic acids from medicinal plants and compared the extraction effectiveness with that of the matrix solid-phase dispersion (MSPD) technique. MSPD is a mode of dSPE in which the sample is directly mixed with the sorbent and the homogeneous mixture is packed in the SPE cartridge and eluted with liquid solvents [104]. dSPE with the use of polyaniline-modified zeolite NaY was applied by Arnnok et al. [105] to extract carbamate, organophosphate, sulfonylurea, pyrethroid, and neonicotinoid from fruit and vegetables. In turn, a modification of dSPE, namely ultrasound-assisted dispersive solid/liquid phase microextraction with the use of a PANI-DBSNa/TiO_2_ composite, was applied to clean up and pre-concentrate calcium-channel blockers (CCBs) in human plasma and urine [106]. Ultrasound-assisted dispersive micro SPE (D-µSPE) based on a CuO nano plate-polyaniline composite was used to isolate insecticides diazinon and imidacloprid from grain samples [107]. In turn, phthalate esters in drinking water and distilled herbal beverages were effectively extracted using a GO/layered double hydroxides/sulfonated PANI composite and ultrasound radiation [108].

#### Magnetic Solid-Phase Extraction (MSPE)

Magnetic solid-phase extraction (MSPE) is a form of dispersive solid-phase extraction in which magnetic particles coated with a sorbent are added into a liquid sample. The analyte is adsorbed/absorbed on the sorbent and the particles are easily separated from the solution by applying an external magnetic field. The simplest nanocomposites were synthesized through oxidative polymerization of aniline in the presence of magnetite Fe_3_O_4_, which is used most widely as the magnetic component, because it has great magnetic properties, low toxicity, and is easily synthesized. Graphene oxide (GO) is readily applied as well [4,103]. PANI-coated Fe_3_O_4_ was applied, e.g., for extraction of methylmercury [109], N-glycopeptides [110], and plastic migrants [111]. Silica [112,113,114], carbon [115,116], magnetic graphene oxide (GO) [117], or their mixture [118,119,120] were often applied. Many other additives, including polypyrrole [121,122], octadecyl-bonded silica [123], and polythiophene [124,125] were also used to improve the physicochemical features of the sorbent or/and modify binding capacity towards specific analytes.

An NiFe2O4@SiO2@PANI-IL nanocomposite in the form of a magnetic effervescent tablet was effective in isolation of the organophosphorus pesticides Methamidophos, Malathion, Parathion, and Diazinon in fruit juice samples (HPLC-DAD) [126]. 

In the SPME variant proposed by Farahmandi et al., simultaneous flows of the sample solution and dispersive magnetic beads (Fe_3_O_4_/PANI) were introduced to the chip in the microfluidics system with magnet using two syringe pumps, and the applicability of the proposed method for pre-concentration of parabens from biological samples was demonstrated [127].

In MSPE, the extraction process can be supported by ultrasound. For example, ultrasound-assisted extraction was used for isolation of polycyclic aromatic hydrocarbons (PAHs) from water by Manousi et al. [118], for extraction of mirtazapine and its metabolites from human urine by Ghorbani et al. [128], and for extraction of antibiotics from milk and infant formula by Shirani et al. [129]. In turn, the CO_2_-effervescence assisted the dispersive μSPE procedure with the use of magnetic-layered double hydroxide (Zn-Al-LDH-Fe_3_O_4_) modified with PANI and a surfactant (DBSNa) and was applied for pre-concentration of heavy metals (Ni, Pb, Co, Cd) from cosmetics [130].

As can be seen, polyaniline-based materials immobilized on magnetic nanoparticles have gained increasing interest and multiple applications in different fields, i.e., for isolation of both inorganic and organic components from various matrices (Table 4).

### 3.4. Stir Bar Sorptive Extraction

Stir bar sorptive extraction (SBSE) developed by Baltussen et al. in 1999 [145] is an alternative to SPME. In this technique, a sorbent-coated magnetic stir bar is used. The stir bar can be immersed in a solution or exposed to the gaseous phase above the liquid or solid sample [4]. However, PANI- based sorbents have minor significance in SBSE, and only few works describe such an approach. Polyaniline/α-cyclodextrin and PANI/hydroxyl multi-walled carbon nanotube composites were used as a covering material in SBSE, which was applied for isolation of polychlorinated biphenyls (PCBs), phenols, and non-steroidal anti-inflammatory drugs from environmental samples [146,147]. A polyaniline–polydimethylsiloxane sol-gel-packed spiral stir bar was used for the extraction of five estrogens from environmental and food samples [148].

## 4. Liquid Chromatography (LC)

It should also be mentioned that Si-PANI has a potential as a stationary phase in chromatographic techniques. So far, it has been applied in non-suppressed ion chromatography to separate different inorganic ions, including chloride, bromide, iodide, nitrate, nitrite, phosphate, thiocyanate, and sulfate [21,40,149,150]. In turn, Taraba et al. [151] prepared and characterized stationary phases based on silica and octadecyl silica modified with PANI and investigated the retention behavior of aniline, phenol, pyridine, toluene, and uracyl on both sorbents using capillary liquid chromatography [151]. Moreover, they exploited Si-PANI for analysis of positional isomers of aminoacetophenone, caffeine, and its demethylated derivatives (theobromine, theophylline) in hydrophilic interaction liquid chromatography (HILIC), normal phase (NP), and reversed phase (RP) modes using a mixed-mode retention mechanism of such a sorbent [152]. Furthermore, metal–organic frameworks (MOFs) modified with chiral polyaniline as a column filling were successfully applied for the separation of 12 chiral compounds, including alcohols, ketones, esters, aldehydes, organic acids, and amines [153]. 

Poly(styrene-divinylbenzene) (PS-DVB) was also used as a solid support for the immobilization of PANI, and the PANI/GO composite coated onto the surface of PS-DVB microspheres had good separation performance for conventional and organic anions [154]. PANI-coated PS-DVB monoliths were applied as the stationary phase for analyses of iodide [155]. Si-PANI was also used in thin layer chromatographic (TLC) separations of amino acids [156] and organic dyes [157,158]. Moreover, PANI used as a thin film covering the inner surface of a fused-silica capillary was used to separate small bioactive peptides in the capillary zone electrophoresis technique [159].

## 5. Conclusions

We have summarized the application of polyaniline (PANI) and polyaniline-based materials in sample pretreatment techniques based on solid-phase extraction. It was clearly shown that PANI composites are still being developed and their utilization in various fields is constantly growing. The physicochemical characterization and application potential of such materials is an interesting topic of scientific research. This is confirmed by the recent number of 40–50 papers published yearly and devoted to the synthesis of new PANI-based sorbents or the new applications of the existing PANI materials in different variants of SPE.

Polymer composites based on PANI have great applications in solid-phase extraction techniques, and significant progress has been achieved in this field taking into account selectivity and stability. Currently, two types of commercially available sorbents have the greatest significance in SPE, including polymeric and silica based fillings. Compared to them, PANI based sorbents are characterized by increased durability, stability in drastic acidic-alkaline conditions, the possibility of reuse, and relatively easy regeneration without loss performance. Moreover, due to various additives, they are highly selective towards specific groups of analytes, and therefore, PANI-based sorbents are a promising alternative to existing materials. They have a differentiated retention mechanism and, hence, a capacity of binding a wide range of components. They were successfully used for pretreatment of samples before determination of inorganic and organic analytes using such modern analytical techniques as GC and HPLC.

Further studies should be focused on enhancement of the selectivity by functionalizing the sorbents, e.g., through tests of various polymerization conditions, introduction of various dopants, or combination with other materials, and on searching for a way to enlarge the specific surface area and pore volume of PANI, which will improve the sorption capability of this compound. 

In the future, new PANI nanocomposites should be developed with the elaboration of effective methods of preparation thereof. Moreover, the range of applications of the known PANI-based materials should be further explored in relation to new analytes to enhance their suitability for the SPE technique. Introduction of large-scale production of SPE sorbents based on polyaniline nanocomposites should be also considered in future.

## Figures and Tables

**Table 1:** Application of polyaniline and polyaniline-based material as sorbents in the SPE/μSPE technique.

Material	Analyte/Matrix	Method	LOD	Ref
Polyaniline (PANI)	phenol (Ph), 2,4,-dimethylPh, 2-chloroPh, 4-chloroPh, pentachloroPh/water	GC–FID	not shown (ns)	[19]
PANI	chlorophenols/water	GC–ECD	3 –110 ng L^−1^	[18]
PANI	polar pesticides and their degradation products/water	MEKC–DAD	0.01–0.5 μg L^−1^	[17]
PANI nanowires	triazine, organochlorine, and organophosphorous pesticides/water	GC–MS	0.07–0.3 ng mL^−1^	[16]
PANI on a stainless steel mesh	polycyclic aromatic hydrocarbons (PAHs)/water samples	GC–FID	0.003–0.01 ng mL^−1^	[20]
PANI nanotubes	2′,7′-dichlorofluorescein/aqueous solution	HPLC–FLD	20 nM	[38]
Silica (Si) covered with PANI	trace elements (Fe, Cu, Ni, Zn, Cd, Mn)/selected medicinal plants	HPIC–UV	2–9 μg L^−1^	[23]
Si/PANI	oleanolic, ursolic, betulinic acid/ *Salvia officinalis*, *Syzygium aromaticum*, *Origanum vulgare*	HPLC–DAD	0.11–0.14 μg mL^−1^	[24]
Si/PANI	alkaloids /*Chelidonium majus* extracts	HPLC–DAD	9–17 ng mL^−1^	[25]
Si/PANI	ions (Na, K, Ca, Mg, Cu, Fe, Ni, Co, Cd, Zn, Mn, Pb)/water	HPIC–UV, AAS	0.01–0.2 μg mL^−1^	[26]
Si/PANI (molecularly imprinted)	benzophenone-4/aqueous media	HPLC–DAD	ns	[39]
Si/PANI modified with Acid Alizarin Violet N and Ag^+^ ion	nitrate, nitrite/commercially available bottled water samples	HPIC–UV	6–10 ng mL^−1^	[40]
Ag-NPs/PANI	furosemide/urine samples	HPLC–UV	7 μg L^−1^	[34]
PANI/polyacrylonitrile (PAN)	Sudan dyes/poultry feed	HPLC–DAD	6–15 μgkg^−1^	[31]
PANI/PAN	non-steroidal anti-inflammatory drug residues/meat and egg	UPLC–MS/MS	0.6–12.2 µg kg^−1^	[29]
PANI/PAN nanofiber mat	fluoroquinolones/water, urine and serum	UPLC–MS/MS	0.016–1.52 μg L^-1^	[41]
PANI/PAN nanofiber mat	non-steroidal anti-inflammatory drugs (NSAIDs)/water	UPLC–MS	0.2–5.0 ng L^−1^	[28]
PANI/PAN nanofiber mat	paracetamol (p), chloramphenicol (c)/pork, chicken, and beef	UHPLC–MS	0.15–0.2 (p), 0.01 µg kg^−1^ (c)	[27]
PANI semi-IPN cryogels	antibiotic residues/honey and water samples	HPLC–UV	17–50 μg kg^−1^	[33]
PANI/multi-walled carbon nanotubes/chitosan cryogel	hydrocarbons/tea and coffee	HPLC–UV	0.005–0.05 μg L^−1^	[42]
PANI/ styrene– divinylbenzene	Fluoxetine(f) and norfluoxetine(n)/plasma	LC–FL	10 (f), 80 ng mL^−1^ (n)*	[36]
PS-DVB / TiO_2_/PANI	myricetin (m) and quercetin (q)/*Epipremnum aureum* rhizome	HPLC–UV/Vis	0.009 (m), 0.004 (q) μg mL^−1^	[30]
Polyurethane/PANI chip	morphine, codeine, papaverine/urine sample	UV-Vis spectrophotometry	0.3–1.4 ng mL^−1^	[37]
2-(hexyloxy) naphthalene-sulfate doped PANI	sulfonamide/milk, and honey samples	HPLC–UV/Vis	9.5–16.5 ng mL^−1^	[35]

*—means that the value is limit of quantification (LOQ).

**Table 2:** Application of polyaniline and polyaniline composites in DI-SPME.

Material	Analyte/Matrix	Method	LOD	Ref
PANI on a platinum wire	phenols/water samples	GC–FID	0.69–3.7 ng mL^−1^	[45]
PANI on a platinum wire	phenol and some of its volatile derivatives/water samples	GC–FID	1.3–12.8 ng mL^−1^	[47]
PANI on a platinum wire	polycyclic aromatic hydrocarbons (PAHs)/water samples	GC–MS	0.1–6 pg mL^−1^	[46]
PANI on a gold wire	anatoxin-a/aqueous samples	GC–MS	11.2 ng mL^−1^	[48]
PANI on a stainless steel wire	organochlorine pesticides (OCPs)/water samples.	GC–ECD	0.1–1.6 ng L^−1^	[49]
PANI on a stainless steel wire	chloro- and nitrobenzenes/water samples	GC–ECD	0.0001–0.01 μg L^−1^	[51]
PANI on a stainless steel wire	phthalates/environmental water samples	GC–FID	0.003–10 μg L^−1^	[50]
PANI on a stainless steel wire	polychlorinated biphenyls (PCBs)/gulf sediment	GC–ECD	0.01–0.05 ng g^−1^	[60]
PANI on basalt fibers	2-hydroxy-4-methoxybenzophenone, phenyl salicylate and 2,4-dihydroxybenzophenone/water samples	HPLC– DAD	0.02–0.05 μg L^−1^	[53]
Silica (Si)/PANI inside a stain-steel needle	polycyclic aromatic hydrocarbons (PAHs) and benzene, toluene, ethylbenzene, xylenes/polluted soil samples	GC–FID	0.001–0.1 ng g^−1^	[61]
Si-PANI fiber coating on a stainless steel wire	PAHs/aqueous samples	GC–MS	2–20 pg mL^−1^	[62]
PANI-polypyrrole on a stainless steel wire	o-xylene, phenol, benzyl alcohol, and methyl benzoate/water samples	GC–FID	ns	[63]
PANI on polyester fiber	volatile organic compounds (VOCs)/lemon juice	GC–FID	ns	[64]
PANI/Ag on optical fibers	bifenthrin (pesticide)/water samples	MALDI TOF–MS	10 ng L^−1^	[65]
PANI on titania nanotubes	UV filters/water samples	HPLC–DAD	0.03–0.05 μg L^−1^	[52]
Fluorinated PANI on a polydimethylsiloxane (PDMS) fiber	PAHs/environmental water samples	GC–FID	0.01–0.1 μg L^−1^	[54]
Fluorinated PANI on a stainless fiber	polychlorinated biphenyls (PCBs)/water samples	GC–µECD	0.05–0.1 ng L^−1^	[55]
PANI doped with polydimethylsiloxane (PDMS) on a stainless steel wire	n-tridecane, n-tetradecane and n-pentadecane/aqueous samples	GC–FID	ns	[66]
PANI doped with PEG and PDMS on a stainless steel wire	phenols (bisphenol A, 4-n-nonylphenol, and 4-tert-octylphenol)/water	HPLC–FL	0.014–0.091 μg L^−1^	[56]
PANI/Graphene(G) on a platinum wire	organochlorine pesticides: heptachlor, aldrin, endrin and p,p’-DDT/water	GC– ECD	3.6 – 11 ng L^−1^	[67]
PANI/graphene oxide (GO)	tetracyclines/milk and water	HPLC–UV	0.32–7.59 μg L^−1^	[68]
Graphenized graphite/PANI	PAHs: phenanthrene, anthracene, fluoranthene, and pyrene/ water	HPLC–UV	0.016 – 0.275 μg L^−1^	[69]
PANI/G on the internal surface of a stainless-steel tube	aldehydes/breath condensate	HPLC–UV/Vis	0.02–0.04 nmol L^−1^	[70]
PANI/ MWCNT on stainless steel	thymol (t), carvacrol (c)/medicinal plants, honey	HPLC–UV	0.6(t), 0.8(c) μg mL^−1^	[71]
PANI, PANI/MWCNTs on a platinum wire	methylenedioxymethamphetamine hydrochloride/water	GC–MS	ns	[59]
PANI/MWCNT/amino-modified metal-organic framework UiO-66 UiO-66-NH_2_ on a stainless steel wire	polycyclic aromatic hydrocarbons (PAHs)/lake water	HPLC–UV/Vis	10 pg mL^−1^	[72]
MWCNT/PANI/PPy/polydimethylsiloxane	pesticides (hexachlorobenzene, chlorothalonil, fipronil, chlorfenapyr)/garlic	GC–MS	0.38 –1.90 ng g^-1^	[73]
aniline and m-amino benzoic acid on a platinum wire	saturated-fatty acids/zooplankton	GC–MS	0.01–6.07 μg L^−1^	[74]
ZnO nanorods on a porous PANI	benzene homologues/water	GC–FID	0.001–0.024 μg L^−1^	[75]
thiolated aniline-analog monomers on the gold surface	PAHs: phenanthrene, anthracene, pyrene, 9,10-dimethylanthracene, benzo[α]anthracene/seawater	GC–FID	0.1–0.32 μg L^−1^.	[76]
Poly(diallyldimethylammonium chloride)/GO)-coated C18 - quartz fiber PANI	acidic pharmaceuticals/fish	HPLC–MS/MS	0.13–7.56 ng g^−1^	[77]
PANI/TiO_2_ carbon nanorods	phthalate esters/water samples	HPLC–DAD	0.01–0.05 μg L^−1^	[78]
Polyoxomolybdate_368_/PANI on a stainless steel wire	amitriptyline, nortriptyline, doxepin/urine and blood	HPLC–UV	0.2 ng L^−1^	[79]
Polycaprolactam/PANI on a stainless steel mesh sheet	angiotensin ΙΙ receptor antagonists (ARA-ΙΙs)/human plasma	HPLC–UV/Vis	0.9–1.8 μg L^−1^	[80]
Sulfonated-PANI/polyacrylonitrile nanofiber mats	fluoroquinolones/various animal-origin foods	UPLC–MS	0.012−0.06 μg·kg^−1^	[81]

**Table 3:** Application of polyaniline and polyaniline composites in HS-SPME.

Material	Analyte/Matrix	Method	LOD	Ref
PANI on a gold wire	aliphatic alcohols/gaseous samples	GC–FID	15–75 ng mL^−1^	[82]
PANI on a gold wire	phenol and 4-chlorophenol/gaseous and aqueous samples	GC–FID	2.8–3.0 ng mL^−1^	[83]
PANI on a stainless steel wire	organoarsenic and organophosphorus compounds/soil	GC–MS	0.006–0.45 ng g^−1^	[84]
PANI on a stainless steel wire	tamoxifen/urine samples	GC–FID	0.51 μg L^−1^	[88]
PANI-coated needle	phthalates/water	GC–FID	8.00–37.48 ng	[86]
PANI -nylon-6 as a nanofiber sheet	chlorobenzenes (CBs)/aquatic media	GC–MS	19–33 ng L^−1^	[87]
PANI-metanilic acid on a platinum	1,4-dioxane/water samples	GC–FID	0.1 ng mL^−1^	[89]
Fluorinated PANI on a platinum wire	benzaldehyde/injectable pharmaceutical formulations	GC–FID	16 ng mL^−1^	[44]
PANI-montmorillonite nanocomposite on a stainless steel wire	phenol (Ph), 4-chloroPh, 2,4-dichloroPh, 4-nitroaniline/water	GC–MS	5–14 pg mL^−1^*	[90]
Metal organic framework-PANI on a plunger needle	chlorobenzenes/aqueous samples	GC–MS	0.1–0.2 ng L^−1^	[91]
PANI/MWCNTs on the interior surface of a stainless steel needle	PAHs/polluted soil samples	GC–FID	0.002–0.02 ng g^−1^	[85]
MWCNTs/PANI on a platinum wire	phenol (P) derivative (2-chloroP, 2,4-dichloroP, 2-methylP, 3-methylP, 2,6-dimethylP, 2-nitroP)/water samples	GC–FID	1.89–65.9 ng L^−1^	[92]
Poly(p-phenylenediamine-co-aniline) on a stainless steel wire	chloro- and methyl- derivatives of benzene/gaseous samples	GC–FID	0.2–0.88 μg L^−1^	[93]
PANI–polypyrrole on a stainless steel wire	esters (i.e., methylanthranilate, ethyl-o-aminobenzoate,)/water	GC–FID	0.05−0.38 μg L^−1^	[94]
PANI-ionic liquid (IL) (1-butyl-3-methylimidazolium hexafluorophosphate) on a steel wire	organochlorine pesticides/lake water, wastewater, sewage	GC–ECD	0.12–0.31 ng L^−1^	[95]
PANI–IL (1-butyl-3-methylimidazolium tetrafluoroborate) on a platinum wire	benzene (B) derivatives (1,3-dimethylB, 1,2-dimethylB, 1,4-dichloroB, 1,2-dichloroB, 1,3,5-trimethylB,)/ water	GC–FID	9.3–48.1 ng L^−1^	[96]
GO/PANI/zinc nanorods/zeolitic imidazolate framework	VOCs/human body odor	GC–MS	4.98–14.8 ng	[97]
o-aminobenzene sulfonic acid /MWCNTs/PANI on a stainless steel wire	2,4-dichlorophenol/aqueous samples	GC–FID	1.30 ng L^−1^	[98]
PANI/ MWCNTs/zeolitic imidazolate frameworks on a stainless steel wire	organic pollutants/aqueous samples	GC–FID	0.3–0.8 ng L^−1^	[99]
metal-organic framework/PANI magnetite/on a steel wire	hexanal and heptanal/human plasma and urine samples	GC–FID	0.001, 0.01 µg L^−1^	[100]
PANI/XAD-2 needle trap device	naphthalene, phenanthrene/air	GC–FID	0.002–0.09 ng L^−1^	[101]

*—means that the value is limit of quantification (LOQ).

**Table 4:** Application of polyaniline and polyaniline composites in dSPE.

Material	Analyte/Matrix	Method	LOD	Ref
	**dSPE**			
Si/PANI	triterpenic acids/*Viscum album*, *Ocimum basilicum*	HPLC–DAD	0.12–0.14 µg mL^−1^	[104]
Si/PANI	benzophenone-type UV filters/environmental water	CE–MS/MS	0.6–200 pg mL^−1^	[131]
PANI/zeolite NaY	carbamate, organophosphate, sulfonylurea, pyrethroid, neonicotinoid/ food, and environmental samples	HPLC–PDA	0.001–1.00 mg L^−1^	[105]
TiO_2_/PANI	Co/food and water samples	GF–AAS	0.036 µg L^−1^	[132]
PANI-DBSNa/TiO_2_	calcium channel blockers/ human plasma and urine	HPLC–DAD	1.5–3.0 ng mL^−1^	[106]
CuO nano plate/PANI	diazinon and imidacloprid/grain	HPLC–DAD	3 and 0.056 µg kg^−1^	[107]
GO/layered double hydroxides/sulfonated PANI	phthalate esters/drinking water, herbal beverages	GC–MS	0.06–0.3 ng mL^−1^	[108]
	**magnetic**			
Fe_3_O_4_/PANI	parabens/fruit juice, sunscreen, and urine samples	HPLC–UV	3.0–25.0 μg L^−1^	[127]
Fe_3_O_4_/PANI	benzodiazepines: lorazepam, nitrazepam/urine and plasma	HPLC–UV	0.2–2.0 μg L^−1^	[133]
Fe_3_O_4_/PANI	N-glycopeptides/standard protein	MALDI–MS	50 fmol	[110]
Fe_3_O_4_/PANI	plastic migrants/jelly samples	HPLC–MS	10.6–17.1 ng L^−1^*	[111]
Fe_3_O_4_/C/PANI	xanthene colorants: erythrosine B, phloxine B, rhodamine B/beverage, fish	HPLC–UV/Vis	0.1–0.5 μg L^−1^	[115]
Fe_3_O_4_/C/PANI	phenol (Ph), 2,4-dichloroPh, 2,4,5-trichloroPh, pentachloroPh bisphenol A/water	GC–MS	2.52–29.7 ng mL^−1^*	[116]
Fe_3_O_4_/SiO_2_/PANI	sudan red I/drinks	HPLC–UV/Vis	0.001 mg L^−1^	[134]
Fe_3_O_4_/SiO_2_/PANI	anabolic androgenic steroids /dietary supplements and external drugs	HPLC–MS	0.001–0.02 μg L^−1^	[112]
Fe_3_O_4_/SiO_2_/PANI	Se, Te/environmental water samples	ICP–MS	5.3 and 1.2 pg mL^−1^	[113]
Fe_3_O_4_/SiO_2_/PANI	chloroPh (2-chloroPh, 4-chloroPH, 2,4-dichloroPh, 2,4,6-trichloropH)/water	HPLC–UV	0.32–0.6 µg L^−1^	[114]
Fe_3_O_4_/GO (graphene oxide)/PANI	polycyclic aromatic hydrocarbons (PAHs) and nitrated PAHs/water	GC–MS	0.01–0.11 ng mL^−1^	[118]
Fe_3_O_4_/GO/PANI	Cd/tea and rice samples	ET–AAS	3.6 ng L^−1^	[119]
GO/PANI	mirtazapine and its metabolites/human urine and water	HPLC–DAD	0.4–1.1 ng mL^–1^	[128]
GO/PANI	Cr(IV)/environmental samples	GF–AAS	5.0 ng L^−1^	[117]
Fe_3_O_4_/SiO_2_/PANI/GO	Bisphenol-A/water samples	UV–Vis	ns	[120]
Fe_3_O_4_/SiO_2_/PANI/GO	Y, La, Ce, Pr, Nd, Sm, Eu, Gd, Tb, Dy, Ho, Er, Tm, Yb, Lu /tea leaves, water	ICP–MS	0.04–1.49 ng L^−1^	[135]
Fe_3_O_4_/SiO_2_/polypyrrole(PPy)/PANI	Ni, Cd, Pb/food samples	FAAS	0.09–1.1ng mL^−1^	[121]
Fe_3_O_4_/PANI-polythiophene	Ni. Cu/soft drinks and spice samples	FAAS	2.8 and 1.2 µg L^−1^	[124]
Fe_3_O_4_/PANI-polythiophene	Co/soft drinks, spice, vegetable, and water samples	FAAS	1.6 μg L^−1^	[125]
PANI/GO/octadecyl-bonded silica (C18-SiO_2_)/Fe_3_O_4_	fluoroquinolones/honey, milk, and egg	HPLC–UV	0.001–0.010 μg L^−1^	[123]
attapulgite/Fe_3_O_4_/PANI	benzoylurea insecticides/ water	HPLC–DAD	0.02–0.43 μg L^−1^	[136]
C/polypyrrole-PANI	furfurals/baby food and dry milk samples	HPLC–UV/Vis	0.3–0.7 μg kg^−1^	[122]
magnetic GO/SiO_2_/PANI/PPy	Cr(III) and Pb(II)/water and food samples	ICP–MS	4.808, 3.401 ng L^−1^	[137]
aluminum-metal organic framework/Fe_3_O_4_/PANI	anti-cancer drugs: imatinib, methotrexate irinotecan/biological and water	HPLC–UV	0.06–0.33 ng mL^−1^	[138]
PANI/dicationic ionic liquid/Fe_3_O_4_	polycyclic aromatic hydrocarbons/environmental water, sludge, and soil	GC–MS	0.0008–0.2086 µg L^−1^	[139]
one-dimensional PANI/Fe_3_O_4_	fluoroquinolones/honey samples	HPLC–FL	0.4–1.4 ng g^−1^	[140]
carboxylate functionalized SPANI/Fe_3_O_4_	fluoroquinolones/spiked milk samples	HPLC–UV	25.8–30.2 ng.g^−1^	[141]
mixed iron hydroxides (MIHs)/PANI	phenols/in soil, drinking water, and fruit.	HPLC–DAD	0.01–0.3 µg L^−1^	[142]
magnetic halloysite/PANI/Fe_3_O_4_	PAHs/beer samples	GC–MS	1.64–14.20 ng L^−1^	[143]
GO/PANI/N-[3-(trimethoxysilyl) propyl]ethylenediamine.	amoxicillin, ampicillin, penicillin G/milk samples, infant formula	HPLC–UV	0.5–0.9 μg L^−1^	[129]
Fe_3_O_4_/SiO_2_/PANI/hydrophilic monomers/bovine albumin	coumarins/rat plasma	HPLC–DAD	0.02–0.05 µg mL^−1^	[144]
layered double hydroxide with PANI/surfactant/Fe_3_O_4_	Ni, Pb, Co, Cd/cosmetics	FL–AAS	0.9–2.1 ng mL^−1^	[130]
NiFe_2_O_4_/SiO_2_/PANI-IL	methamidophos, malathion, parathion, diazinon/fruit juice	HPLC–DAD	0.06–0.17 μg L^−1^	[126]

*—means that the value is limit of quantification (LOQ).

## Data Availability

Not applicable.

## Abbreviations

AAS	atomic absorption spectrometry
DAD	diode array detector
DI-SPME	direct immersion SPME
dSPE	dispersive solid-phase extraction
ECD	electron capture detector
FAAS	flame AAS
FID	flame ionization detector
FL	fluorescence detector
GC	gas chromatography
GO	graphene oxide
HPIC	high-pressure ion chromatography
HPLC	high-performance liquid chromatography
HS-SPME	headspace SPME
ICP	inductively coupled plasma detector
LC	liquid chromatography
IL	ionic liquid
MALDI	matrix assisted laser desorption ionization
TOF	time of flight detector
MEKC	micellar electrokinetic chromatography
MNPs	magnetic nanoparticles
MS	mass spectrometry
MSPE	magnetic solid-phase extraction
MWCNT	multiwall carbon nanotubes
NPs	nanoparticles
PANI	polyaniline
PAHs	polycyclic aromatic hydrocarbons
PPy	polypyrrole
PS-DVB	poly (styrene-divinylbenzene)
SBSE	stir bar sorptive extraction
SBSE	stir-bar sorptive extraction
Si-PANI	silica gel covered with polyaniline
SPE	solid-phase microextraction
SPME	solid-phase microextraction
UHPLC	ultra high performance/pressure liquid chromatography
UPLC	ultra performance liquid chromatography
UV-VIS	ultraviolet visible spectroscopy
μSPE	micro solid-phase microextraction
